# Immunotherapy response and resistance in patients with advanced uveal melanoma: a retrospective cohort study

**DOI:** 10.1007/s10238-024-01497-8

**Published:** 2024-10-01

**Authors:** Alexander Maurer, Giulio Clerici, Jan A. Schaab, Phil F. Cheng, Daniela Mihic-Probst, Cäcilia Mader, Michael Messerli, Martin W. Huellner, Reinhard Dummer, Florentia Dimitriou

**Affiliations:** 1 Department of Nuclear Medicine, University Hospital of Zurich, University of Zurich, Zurich, Switzerland; 2 Department of Dermatology, University Hospital of Zurich, University of Zurich, Raemistrasse 100, 8091 Zurich, Switzerland; 3 Department of Oncology, Geneva University Hospital, Geneva, Switzerland; 4 Institute for Pathology and Molecular Pathology, University Hospital Zurich, University of Zurich, Zurich, Switzerland

**Keywords:** Uveal melanoma, Immunotherapy, Immune checkpoint inhibitors, Tebentafusp

## Abstract

Metastatic uveal melanoma (mUM) is associated with poor prognosis. Ipilimumab/nivolumab has shown antitumor efficacy in phase II studies. Tebentafusp resulted in longer overall survival (OS) compared to investigator`s choice in a phase III study. We sought to describe the radiological response patterns of mUM patients treated with immunotherapy. Patients with mUM treated with ipilimumab/nivolumab and tebentafusp between July 2018 and December 2022, with available radiological assessment per RECISTv1.1 and/or imPERCIST5, were retrospectively identified and included. Progression-free survival (PFS) and OS rates, liver-specific response and pathological assessment in available liver biopsies were evaluated. In the ipilimumab/nivolumab group, median PFS (mPFS) was 2.9 months (95% CI 2.2–28.6) and mOS 28.9 months (95% CI 12.7-NR). Complete (CMR) and partial (PMR) metabolic response per imPERCIST5, and partial response (PR) per RECISTv1.1 were associated with longer PFS and OS by trend, compared to morphologically and metabolically stable or progressive disease. In the tebentafusp group, mPFS was 2.7 months (95% CI 2.2–3) and mOS 18.6 months (95% CI 11.5-NR). PMR and PR were associated with longer PFS by trend. In both treatments, the overall treatment response was associated with the radiological response at the liver site. In available liver tumor biopsies, differences in pathological and radiological responses were noted. ImPERCIST5 and RECIST v1.1 are valuable tools in the radiological response assessment, but both methods display limitations. Accurate biomarkers to stratify patients at risk for disease progression and future translational studies to investigate mechanisms of response and resistance are required.

**Supplementary Information:**

The online version contains supplementary material available at 10.1007/s10238-024-01497-8.

## Introduction

Uveal melanoma (UM) is a rare disease arising from the melanocytes of the uveal tract [1]. It represents approximately 5% of all melanoma diagnoses [2], but comprises the most frequent intraocular malignancy in adults [3, 4]. Approximately 50% of patients diagnosed with UM will develop metastatic disease, with the liver being the most common site. Metastatic UM (mUM) has been traditionally associated with a poor prognosis, with median overall survival (mOS) of 6–12 months and 3-year overall survival rate of less than 10% [5, 6]. This comparably high mortality has been partially attributed to its distinct biology, with high rates of distant metastases [7] and absence of the common genetic driver mutations detected in cutaneous melanoma (CM) [8]. In fact, UM commonly harbors *GNAQ* or *GNA11* oncogenic mutations [9], which can be also detected in uveal nevi, whereas loss of function mutations in *BAP1* are associated with a higher frequency of metastases [10]. Furthermore, UM is characterized by a low tumor mutational burden (TMB), low expression of PD-L1 in primary and metastatic tumor sites [11, 12] and an immunosuppressive tumor microenvironment [13]. This distinct biology also contributes to its low immunogenicity.

The advent of immunotherapy has dramatically changed the treatment landscape of advanced cutaneous melanoma, but this paradigm does not translate into the treatment of advanced UM, where the efficacy of immunotherapy is limited. Clinical trials of single-agent immune checkpoint inhibitors (ICIs) have demonstrated low overall response rates (ORR) of 0–3.6% and mOS between 6.8 and 7.6 months for single-agent ipilimumab and anti-PD1, respectively [14, 15]. Two phase II clinical trials investigating the efficacy of combined ICIs with ipilimumab/nivolumab showed a modest improvement in OS; in a small phase II study of 35 patients with mUM, systemic treatment with ipilimumab/nivolumab yielded an ORR of 18% with median progression-free survival (mPFS) of 5.5 months (95% CI 3.4–9.5 months) and mOS 19.1 months (95% CI 9.6-NR months) [16]. Of note, the median duration of response (DoR) in responders was 12.1 months (range 2.8–43 months) and after a median follow-up (mFU) of 13 months, three patients had ongoing response. In the first-line setting, combined ICIs (ipilimumab/nivolumab) in 52 patients with mUM showed similar efficacy with ORR 11.5%, whereas stable disease (SD) was the most common treatment outcome with disease control rate (DCR) 63.5% [17]. In the same study, in addition to the conventional response patterns, ten patients with pseudoprogression were identified. Altogether, these data demonstrate that ICIs have modest efficacy in the treatment of mUM, with heterogeneous and often short-lived responses that merit further investigation.

Recently, tebentafusp, a first-in-class immunotherapy that engages T-cells with glycoprotein 100 (gp100) on melanoma cells, demonstrated an OS benefit in a phase III clinical trial of patients with HLA-A*02:01-positive mUM and gained regulatory approval [18]. The results of the phase III study reported significant improvement in mOS for patients receiving tebentafusp compared to investigators choice treatment [21.7 months (95% CI 18.6-28.6) vs. 16 months (95% CI 9.7-28.6)], yielding a risk reduction of 49% for death (HR 0.51, *p*<0.001) [18]. Despite this benefit in OS, there was no significant improvement in PFS and ORR and a survival benefit was also observed in patients with best overall response (BOR) progressive disease (PD), suggesting that tebentafusp induces a change in the tumor microenvironment that might not be measurable using conventional radiological techniques.

In our study, we sought to evaluate the radiological response patterns of mUM patients treated with immunotherapy, including both ipilimumab/nivolumab and tebentafusp. As UM preferentially metastasizes to the liver, and immunotherapy has proven less efficient in this organ, the hepatic treatment response was also evaluated.

## Methods

### Study population

In this retrospective cohort study we included patients with history of UM and documented metastatic disease that was treated with immunotherapy at the Dermatology Department of the University Hospital of Zurich between July 2018 and December 2022. Patients were identified from the institution’s database. Patient and disease characteristics were derived from electronical medical records. Immunotherapy regimens included either combined ICIs with ipilimumab/nivolumab or tebentafusp. Patients receiving ipilimumab/nivolumab underwent standard treatment with ipilimumab (3mg/kg every 3 weeks) and nivolumab (1mg/kg every 3 weeks). For tebentafusp, patients were treated either as part of a clinical trial (NCT03070392) or through an early access program. Further inclusion criteria included histologically confirmed metastases from UM and available radiological assessment with computer tomography (CT), magnetic resonance imaging (MRI) of the liver or 2-[^18^F]-fluorodeoxy-d-glucose whole-body positron emission tomography/computed tomography (FDG-PET/CT) at baseline (before treatment start) and after 12 weeks (+/− four weeks). In addition, the presence of at least one target lesion meeting the Response Criteria In Solid Tumors version 1.1 (RECISTv1.1) for CT and MRI [19] or immunotherapy-modified Positron Emission Tomography Response Criteria In Solid Tumors (imPERCIST5) for FDG-PET/CT [20, 21] was required at baseline. Exclusion criteria were: non-evaluable radiological disease at baseline and administration of concurrent liver-directed treatment between baseline and first radiological assessment after treatment initiation.

Patients' baseline characteristics, including age, sex, mutational status, previous treatments, number and sites of metastatic organs involved and lactate dehydrogenase (LDH) levels at treatment start, as well as laboratory liver parameters, were collected. Disease features, treatment characteristics and outcome during immunotherapy were additionally assessed and analyzed. Disease outcome and landmark PFS and OS were reported according to CT, MRI or FDG-PET/CT response. In a subset of patients treated with tebentafusp, pathological assessment from available liver biopsies was compared with their radiological assessment at the baseline and during treatment. Ethics approval was obtained from the local ethics committee (KEK Zürich, BASEC-Nr. 2022-00551), and the study was conducted in compliance with ICH-GCP rules and the Declaration of Helsinki.

### Response evaluation

Disease assessment and treatment response evaluation was performed by contrast-enhanced CT of the chest and abdomen or whole-body FDG-PET/CT. If available, liver metastases were evaluated with a dedicated MRI of the liver. The treatment efficacy in CT or MRI was assessed using RECIST v1.1 [19], and the metabolic response in FDG-PET/CT was assessed with imPERCIST5 [20, 21]. Response assessment by CT and MRI was classified as complete response (CR), partial response (PR), stable disease (SD) and progressive disease (PD). Response assessment by FDG-PET/CT was classified as complete metabolic response (CMR), partial metabolic response (PMR), stable metabolic disease (SMD) or progressive metabolic disease (PMD). To assess the response of liver metastases in CT, MRI and FDG-PET/CT, at least two liver lesions were selected for evaluation. Imaging examinations were collected at the baseline and at 12 weeks (+/− four weeks) from treatment start, according to the standard of care. Two independent physicians, who were dually board-certified in radiology and nuclear medicine and were blinded to the patient data, reviewed all CT, MRI and FDG-PET/CT images. In case of discrepancy, a consensus decision was reached with individual case discussion.

### Statistical analysis

Patient and disease characteristics were summarized using descriptive analysis. Categorical variables were calculated by frequencies, and median values and ranges were used for continuous variables. The duration of survival was calculated using the Kaplan–Meier method, and comparisons between the curves were made using the exact log-rank test. Progression events were based on CT and FDG-PET/CT correlation, based on the previously mentioned response criteria. PFS was calculated from treatment start until disease progression, while OS was calculated from treatment start until death, or last date of follow-up if patients were alive. Patients with no progressive disease or who were alive at data collection cutoff (May 2023), were censored. The follow-up period was calculated from treatment start to the date of last visit or death using the inverse Kaplan–Meier method. Survival performance based on type of responses (CT or FDG-PET/CT) was tested using the Cox proportional hazard model. Log-rank test was used to assess the survival differences between groups. All statistical tests were two-sided and *p*-values <0.05 were considered statistically significant. Statistical analyses were conducted using R (version 3.6.1).

## Results

### Patient characteristics

Fourteen patients were treated with combined ICIs (ipilimumab/nivolumab). Twenty-two patients received tebentafusp. Demographics and baseline characteristics of the two groups are summarized in Table 1. In the ICI cohort, the median age was 58 years (range 22–72) and ten (71%) patients were male. Tumor mutational status was available for 12 (86%) patients. Thereof four (29%) had a *GNA11*, and seven (50%) a *GNAQ* mutation. At the time of the treatment initiation, nine (64%) patients had both hepatic and extrahepatic metastases; the most common extrahepatic metastatic sites were lung (*n*=7, 50%), lymph nodes (*n*=6, 43%), soft tissue (*n*=5, 36%) and bone (*n*=4, 31%). Disease stage at treatment start was predominantly stage IV AJCCv8 M1a (*n*=6, 46%) and M1b (*n*=4, 31%), whereas three (23%) patients had stage IV M1c disease. Five (36%) patients were treatment naïve; of the nine (64%) patients that had received one or more previous treatment lines, two had been treated with single-agent anti-PD1 and six with tebentafusp. At the time of the treatment initiation, alkaline phosphatase was normal in seven (50%) patients and five (36%) patients had elevated LDH serum levels.

**Table 1:** Demographics and baseline characteristics of patients with metastatic uveal melanoma (mUM) treated with immune checkpoint inhibitor (ICI) treatment ipilimumab/nivolumab or tebentafusp

Characteristic	ICI N = 14	Tebentafusp *N* = 22
*Age at primary diagnosis*		
Median (Range)	58 (22–72)	57 (18–75)
*Sex*		
Female	4 (29%)	8 (36%)
Male	10 (71%)	14 (64%)
*Metastatic stage (AJCC, 8th edition)*		
M1	0	1 (5%)
M1a	6 (46%)	5 (23%)
M1b	4 (31%)	15 (68%)
M1c	3 (23%)	1 (5%)
Unknown	1	0
*Number of organs involved*		
1	5 (36%)	7 (32%)
2	2 (14%)	4 (18%)
≥ 3	7 (50%)	11 (50%)
*Sites of metastatic disease*		
Hepatic only	5 (36%)	7 (32%)
Hepatic and extrahepatic	9 (64%)	15 (68%)
Liver disease		
Unilobular	3 (21%)	3 (14%)
Multilobular	11 (78%)	19 (87%)
*Extrahepatic disease*		
Bone	4 (31%)	6 (27%)
Lung	7 (50%)	9 (41%)
Nodal	6 (43%)	10 (45%)
Soft tissue	5 (36%)	10 (45%)
Brain	0	1 (5%)
Other	7 (50%)*	5 (23%)**
*Mutation status*		
* GNA11* only	2 (14%)	2 (9%)
* GNAQ* only	3 (21%)	6 (27%)
* GNA11* and *SF3B1*	1 (7%)	1 (5%)
* GNAQ* and *SF3B1*	1 (7%)	1 (5%)
* GNA11* and *BAP1*	1 (7%)	5 (23%)
* GNAQ* and *BAP1*	3 (21%)	2 (9%)
Not tested	2 (14%)	5 (23%)
*Prior lines of therapy*		
None	5 (36%)	14 (63%)
1	7 (50%)	2 (9%)
≥ 2	2 (14%)	5 (23%)
*LDH level at treatment start*		
Normal	6 (55%)	10 (45%)
Elevated	5 (36%)	12 (54%)
Unknown	3	0
*GGT at treatment start*		
Normal	5 (36%)	11 (50%)
Elevated	1 (7%)	6 (27%)
Unknown	8 (57%)	5 (23%)
*ALP at treatment start*		
Normal	5 (36%)	15 (68%)
Elevated	5 (36%)	7 (32%)
Unknown	4 (29%)	0 (0%)
*ALAT at treatment start*		
Normal	7 (50%)	14 (64%)
Elevated	3 (21%)	8 (36%)
Unknown	4 (29%)	0 (0%)
*ASAT at treatment start*		
Normal	6 (43%)	16 (73%)
Elevated	4 (28%)	6 (27%)
Unknown	4 (29%)	0 (0%)
*Liver-directed therapy*		
Operation	1 (7%)	1 (5%)
Radiotherapy	1 (7%)	2 (9%)
None	11 (79%)	18 (82%)

*Other organs included: adrenals (*n*=3), thyroid gland (*n*=2), pancreas (*n*=1) and spleen (*n*=1).

**Other organs included: kidney (*n*=2), thyroid gland (*n*=2), pancreas and kidney (*n*=1).

AJCC, American Joint Committee on Cancer; ALAT, alanine aminotransferase; ALP, alkaline phosphatase; ASAT, aspartate aminotransferase; BAP1, ubiquitin carboxyl-terminal hydrolase BAP1; GGT, gamma-glutamyl transferase; GNA11, guanine nucleotide-binding protein subunit alpha-11; GNAQ, guanine nucleotide-binding protein subunit alpha q; ICI, immune checkpoint inhibitor; LDH, lactate dehydrogenase; SD, standard deviation; SF3B1, splicing factor 3b subunit 1

In the tebentafusp group, the median age at disease diagnosis was 57 years (range 18–75) and 14 (64%) patients were male. Mutational status was *GNA11* and *GNAQ* in eight (36%) and nine (41%) patients, respectively. Hepatic and extrahepatic metastases were present in 15 (68%) patients, and the most frequent extrahepatic metastatic sites were lymph nodes (*n*=10, 45%), soft tissue (*n*=10, 45%), lung (*n*=9, 41%) and bone (*n*=6, 27%). At the time of treatment initiation, most patients had stage IV AJCCv8 M1b disease (*n*=15, 68%), whereas five (23%) patients had M1a and one (5%) patient M1c disease stage. In this treatment cohort, 14 (63%) patients were treatment naïve. Seven (32%) patients were pretreated with ipilimumab/nivolumab, and five (23%) patients had ≥2 previous treatment lines. Alkaline phosphatase was normal in 15 (68%) patients, and 12 (54%) patients had elevated LDH serum levels at treatment start.

### Treatment characteristics

In the ICI group, median follow-up (mFU) was 26.6 months (range 0.5–31.2 months) and at the time of data cut-off (June 2023), six (43%) patients deceased due to disease progression. Overall, five (36%) patients completed all four induction treatment cycles. The median time on treatment was 77 days (range 16–458 days), and the median number of infusions received was five (range 1–13). Reasons for treatment discontinuation included treatment toxicity (*n*=7, 50%), disease progression (*n*=6, 43%) and patient's choice (*n*=1, 7%). Toxicities that led to treatment discontinuation included immune-related hepatitis (*n*=3), pancreatitis (*n*=3), colitis (*n*=2), thyroiditis (*n*=2), nephritis (*n*=1) and myocarditis (*n*=1).

In the tebentafusp group, mFU was 20.1 months (range 1.0–48.2). At the time of data collection, ten (45%) patients deceased due to melanoma. The median time on treatment was 316 days (range 25–947 days), and the median number of infusions received was 44 (range 4–93). Reason for treatment discontinuation was disease progression in all 22 (100%) patients.

### Overall treatment efficacy

**Fig. 1:**
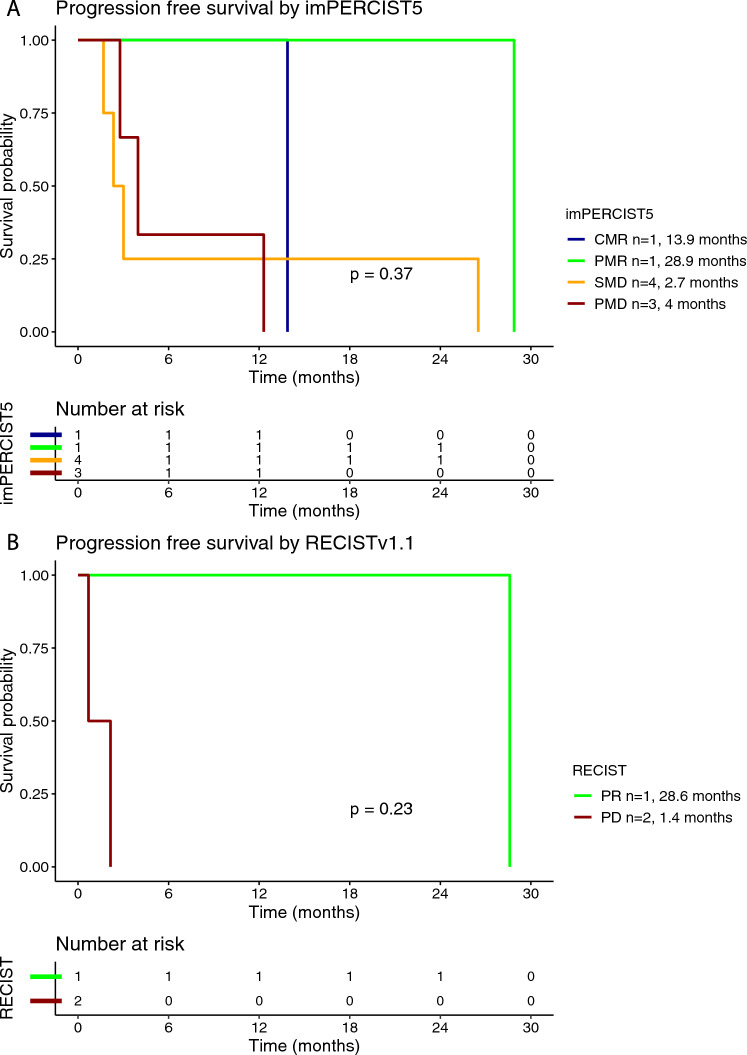
Kaplan–Meier curve for progression-free survival (PFS) according to the best overall response (BOR) using imPERCIST5 (**A**) and RECISTv1.1 (**B**) criteria in patients treated with ipilimumab/nivolumab

**Fig. 2:**
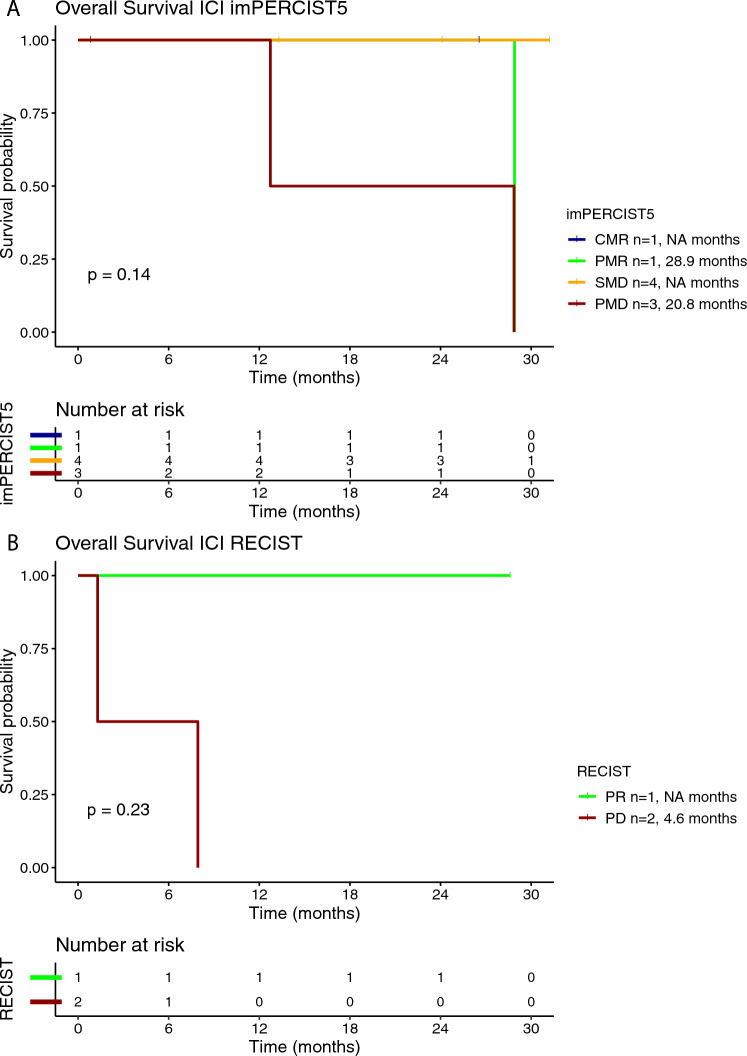
Kaplan–Meier curve for overall survival (OS) according to the best overall response (BOR) using imPERCIST5 (**A**) and RECISTv1.1 (**B**) criteria in patients treated with ipilimumab/nivolumab

Twelve (86%) out of fourteen patients treated with ipilimumab/nivolumab had available radiological images that were eligible for a comprehensive radiological assessment. Treatment efficacy for the BOR was evaluated using imPERCIST5 in nine (75%) patients as shown in Table 2. RECISTv1.1 (RECIST) was used for the radiological evaluation of the treatment efficacy in three (25%) patients. Overall, objective response was observed in three (25%) out of 12 patients; one (8%) patient had a CMR as BOR and developed acquired resistance after 12.9 months of response. Disease control was achieved in seven (58%) patients. The median PFS for the patients treated with ipilimumab/nivolumab was 2.9 months (95% CI 2.2–28.6 months) (Supplementary Figure 1A). Patients with CMR or PMR as BOR per imPERCIST5 had longer mPFS by trend, which was 13.9 months and 28.9 months, respectively. Patients with SMD and PMD had mPFS of 2.7 months and 4 months, respectively (Fig. 1A). Similarly, patients with PR per RECIST had longer mPFS by trend, which was 28.6 months, whereas the respective value in patients with PD was 1.4 months (Fig. 1B). Elevated LDH at treatment initiation was associated with shorter PFS, compared to normal LDH (0.8 months vs. 3.4 months, respectively) (Supplementary Figure 2). The median OS for the patients treated with ipilimumab/nivolumab was 28.9 months (95% CI 12.7-NR months) (Supplementary Figure 1B). In the subgroup analysis for the OS rate stratified by BOR per imPERCIST5, median OS was by trend longer in patients with CMR (mOS not reached) and PMR (mOS 28.9 months), compared to SMD (mOS not reached) and PMD (mOS 20.8 months) (Fig. 2A). Similarly, the median OS was by trend longer in patients with PR per RECIST (mOS not reached), compared to PD (mOS 4.6 months) (Fig. 2B). The median OS was by trend shorter in patients with elevated baseline LDH (mOS 7.9 months) compared to normal baseline LDH (mOS 28.9 months) (Supplementary Figure 3).

**Table 2:** Treatment efficacy for best overall response (BOR) and best treatment response at the liver based on imPERCIST5 and RECISTv1.1 response criteria in patients with available radiological response assessment (ICI; 12 patients for BOR and 9 for best treatment response at liver site)

Characteristic	ICI	Tebentafusp
*Total number of patients with available radiological assessment*	*N* = 12	*N* = 18
***Best overall response (BOR) rate***		
*imPERCIST5*		
CMR	1 (8%)	0
PMR	1 (8%)	2 (11%)
SMD	4 (33%)	3 (17%)
PMD	3 (25%)	7 (39%)
*RECISTv1.1*		
CR	0	0
PR	1 (8%)	1 (6%)
SD	0	1 (6%)
PD	2 (17%)	4 (22%)
***Best treatment response at liver***		
*Total number of patients with available radiological assessment*	*N* = 9	*N* = 12
*imPERCIST5*		
CMR	1 (11%)	0
PMR	0	2 (17%)
SMD	4 (44%)	3 (25%)
PMD	4 (44%)	7 (58%)
*RECISTv1.1*		
CR	1 (11%)	0
PR	1 (11%)	2 (17%)
SD	1 (11%)	6 (50%)
PD	6 (67%)	4 (33%)

CMR, complete metabolic response; CR, complete response; ICI, immune checkpoint inhibitor; PD, progressive disease, PMD, progressive metabolic disease; PMR; partial metabolic response; PR; partial response; SD, stable disease; SMD, stable metabolic disease

**Fig. 3:**
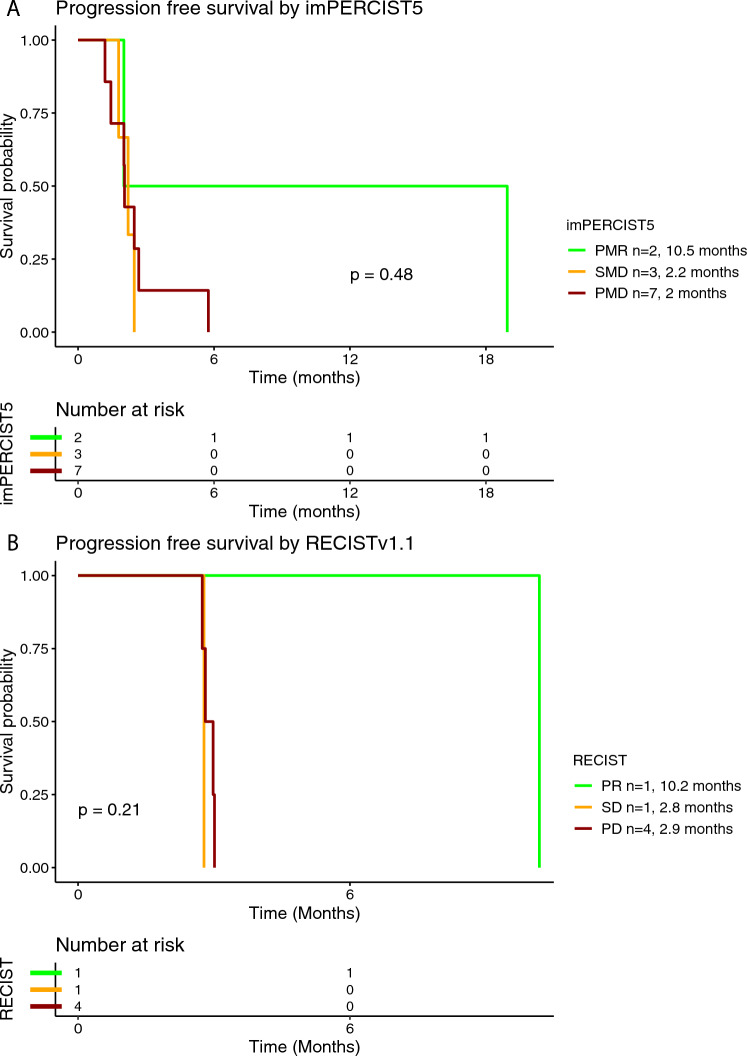
Kaplan–Meier curve for progression-free survival (PFS) according to the best overall response (BOR) using imPERCIST5 (**A**) and RECISTv1.1 (**B**) criteria in patients treated with tebentafusp

**Fig. 4:**
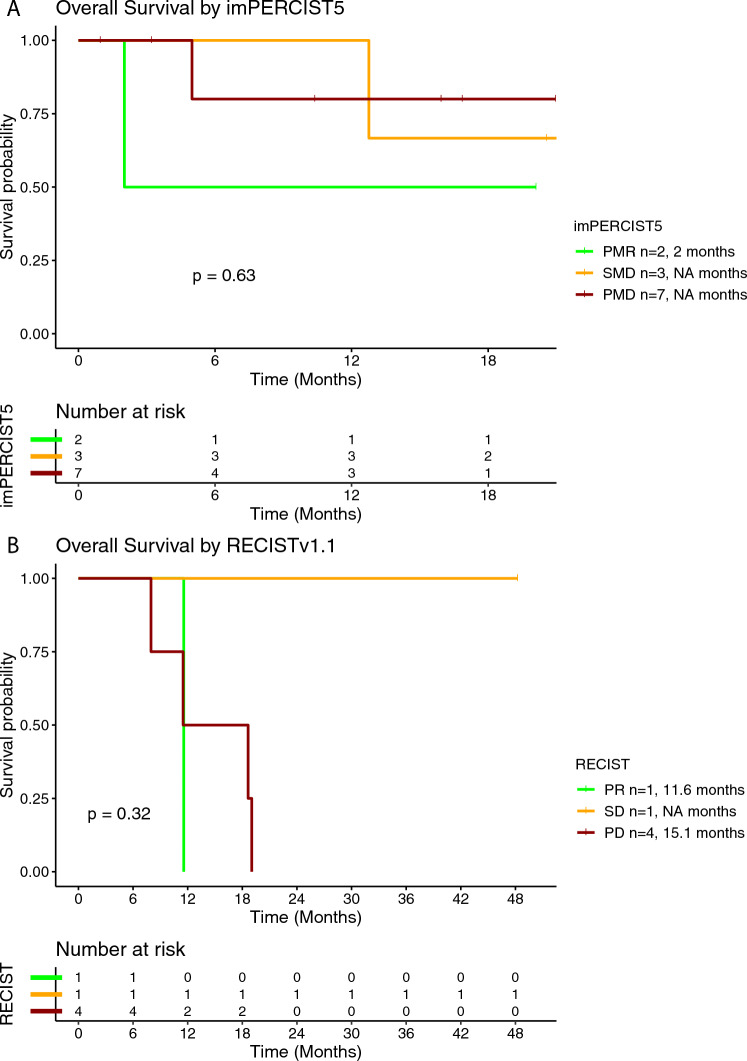
Kaplan–Meier curve for overall survival (OS) according to the best overall response (BOR) using imPERCIST5 (**A**) and RECISTv1.1 (**B**) criteria in patients treated with tebentafusp

In the tebentafusp group, 18 (82%) out of 22 patients were eligible for additional radiological assessment, whereof 12 (67%) were evaluated using imPERCIST5 criteria and six (33%) using RECIST criteria (Table 2). Objective response was achieved in three (17%) patients and disease control in seven (39%) patients. The median PFS for the patients treated with tebentafusp was 2.7 months (95% CI 2.2-3) and the median OS 18.6 months (95% CI 11.5-NR) (Supplementary Figure 4A and B). In the subgroup analysis for the PFS rate stratified by BOR per imPERCIST5, median PFS was by trend longer in patients with PMR (mPFS 10.5 months), compared to SMD (mPFS 2.2 months) and PMD (mPFS 2 months) (Fig. 3A). Similarly, median PFS was by trend longer in patients with PR per RECIST (mPFS 10.2 months), compared to SD (mPFS 2.8 months) and PD (2.9 months) (Fig. 3B). Median OS was 2 months in patients with PMR per imPERCIST5 and not reached in patients with SMD and PMD (Fig. 4A**)**. In the response assessment per RECIST, mOS was 11.6 months in patients with PR, not reached in patients with SD and 15.1 months in patients with PD (Fig. 4B). Median PFS was 2.8 months in patients with normal baseline LDH and 2.6 months in patients with elevated baseline LDH (Supplementary Figure 5). Similarly, median OS was 11.6 months in patients with normal LDH and 19 months in patients with elevated LDH at the time of treatment initiation (Supplementary Figure 6).

### Liver-specific treatment efficacy

**Fig. 5:**
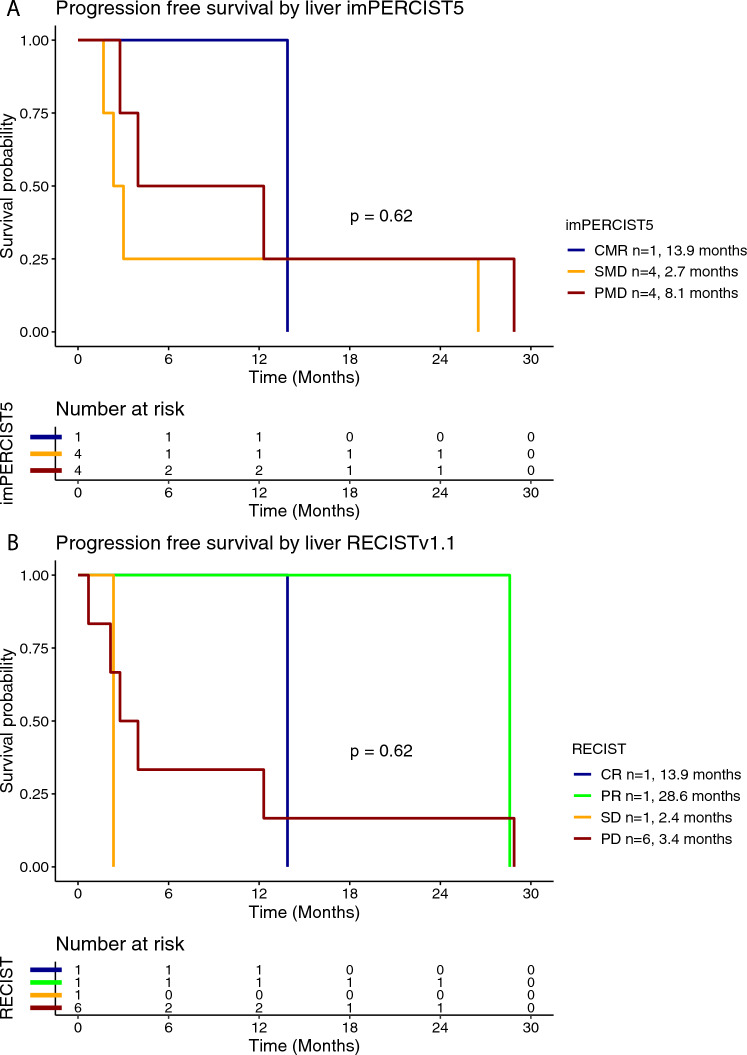
Kaplan–Meier curve for progression-free survival (PFS) according to best treatment response at the liver using imPERCIST5 (**A**) and RECISTv1.1 (**B**) criteria in patients treated with ipilimumab/nivolumab

**Fig. 6:**
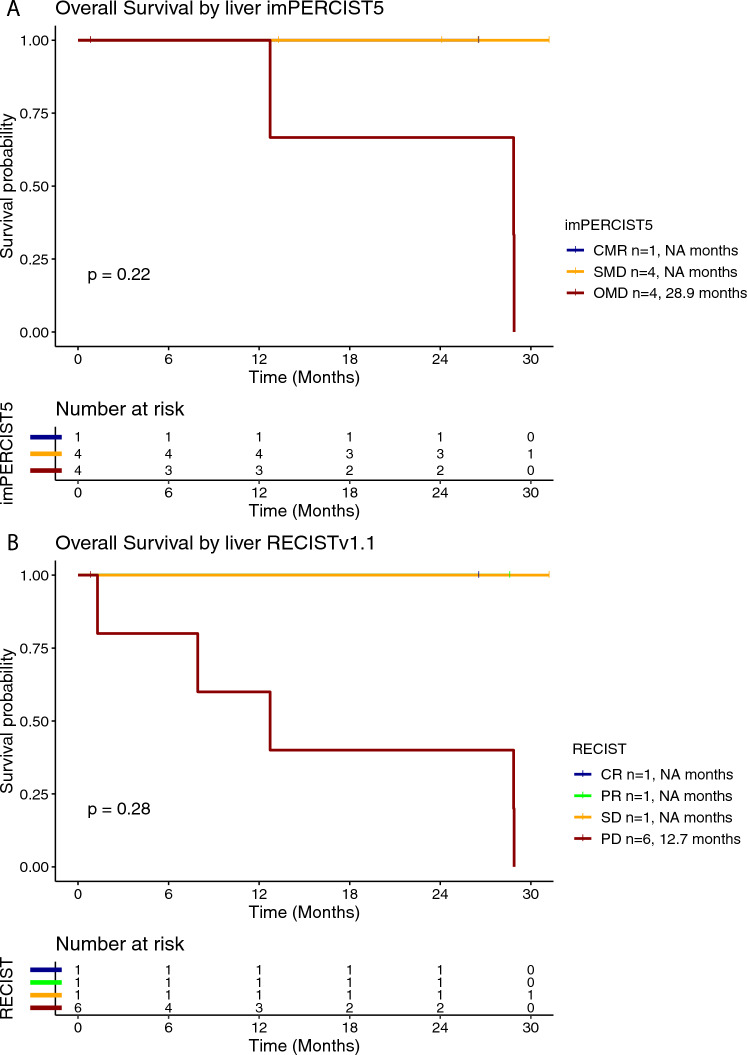
Kaplan–Meier curve for overall survival (OS) according to best treatment response at the liver using imPERCIST5 (**A**) and RECISTv1.1 (**B**) criteria in patients treated with ipilimumab/nivolumab

Of the 14 patients with liver metastases that received ipilimumab/nivolumab, evaluable liver lesions according to imPERCIST5 criteria and RECIST criteria were present in nine (64%) patients (Table 2). In patients with both imPERCIST5 and RECIST liver disease assessment, the liver-specific morphological and metabolic responses were overall associated. Furthermore, the liver-specific treatment efficacy was associated with the overall treatment efficacy. In the subgroup analysis stratified according to the BOR at the liver site, mPFS in patients with CMR at the liver site was 13.9 months, whereas the respective values for SMD and PMD were 2.7 months and 8.1 months, respectively (Fig. 5A). Similarly, there was a trend for longer PFS in patients CR (mPFS 13.9 months) and PR (mPFS 28.6 months), compared to SD (mPFS 2.4 months) and PD (mPFS 3.4 months) as their best response at the liver site (Fig. 5B). Median OS per imPERCIST5 at the liver site was 28.9 months in patients with PMD and not reached for patients with CMR and SMD (Fig. 6A). Accordingly, mOS per RECIST at the liver site was 12.7 months in patients with PD and not reached in patients with CR, PR and SD (Fig. 6B).

**Fig. 7:**
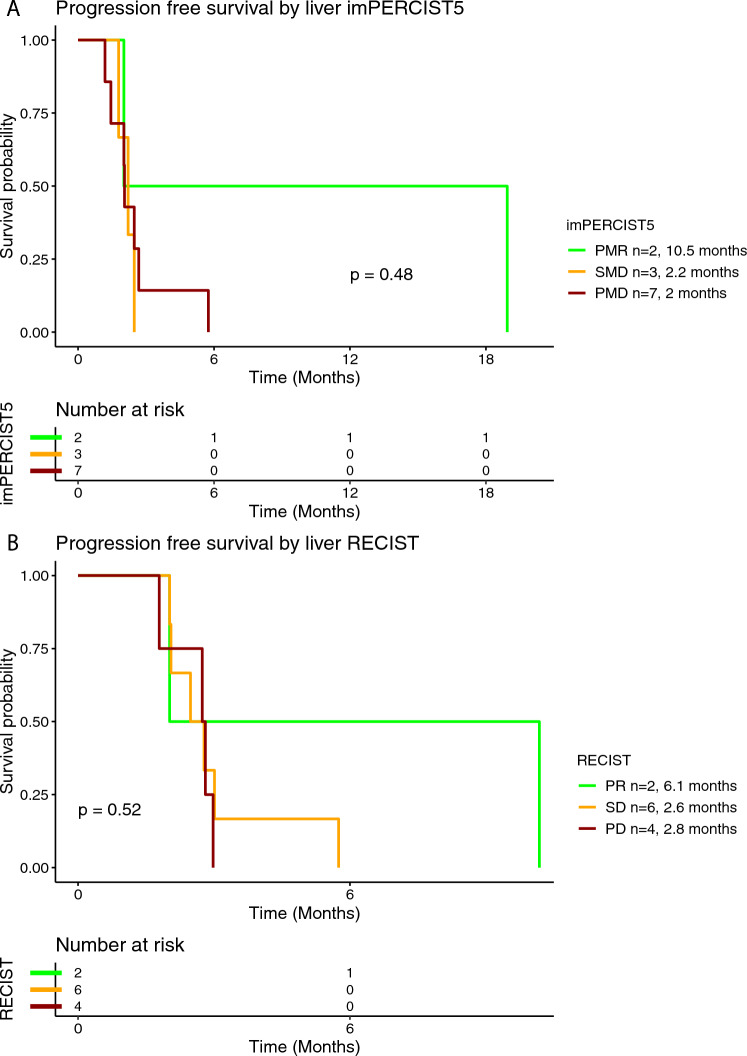
Kaplan–Meier curve for progression-free survival (PFS) according to best treatment response at the liver using imPERCIST5 (**A**) and RECISTv1.1 (**B**) criteria in patients treated with tebentafusp

**Fig. 8:**
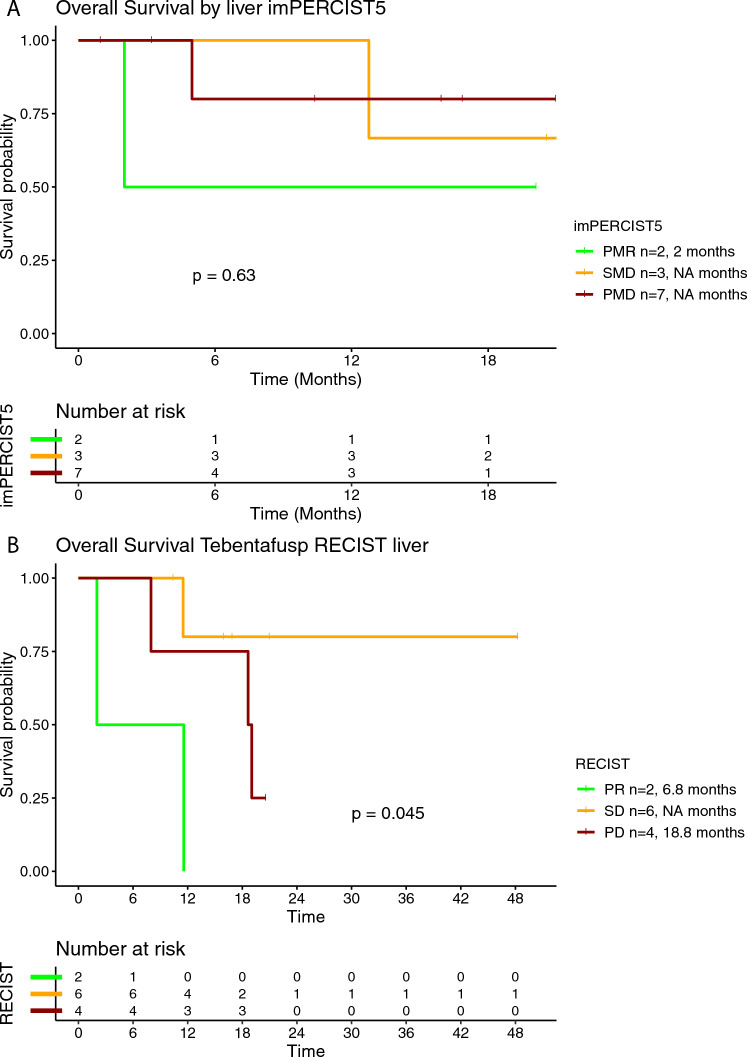
Kaplan–Meier curve for overall survival (OS) according to best treatment response at the liver using imPERCIST5 (**A**) and RECISTv1.1 (**B**) criteria in patients treated with tebentafusp

In the tebentafusp group, 12 (55%) patients were evaluated for their liver-specific response according to imPERCIST5 and 12 (55%) according to RECIST criteria (Table 2). Of note, four patients with both radiological assessments at the liver site and PMD as their best liver response on imPERCIST5, had SD on liver-specific assessment per RECIST. Additionally, one patient with PD on liver-specific assessment per RECIST had SMD on liver-specific assessment per imPERCIST5. Overall, the liver-specific treatment efficacy was associated with the overall treatment efficacy by tebentafusp as well, although one patient with PD as best overall response achieved SD in the liver-specific assessment. In the subgroup analysis stratified according to the BOR at the liver site, mPFS in patients with PMR at the liver site was 10.5 months, and the respective values for SMD and PMD were 2.2 months and 2 months, respectively (Fig. 7A). Similarly, there was a trend for longer PFS in patients with PR at the liver site; mPFS was 6.1 months in these patients, whereas in patients with SD and PD at the liver site, mPFS was 2.6 months and 2.8 months, respectively (Fig. 7B). Median OS per imPERCIST5 at the liver site was 2 months in patients with PMR and not reached for patients with SMD and PMD (Fig. 8A). Accordingly, median mOS per RECIST at the liver site was 6.8 months in patients with PR, not reached in patients with SD and 18.8 months in patients with PD (Fig. 8B).

### Pathological response assessment

Five patients treated with tebentafusp had a pathological response assessment of their liver metastases at the baseline (before treatment initiation) and during treatment, which was concurrent to the radiological assessment. Two patients with PMD and SMD as best radiological response at the liver site showed no pathological response, with histologically isolated tumor-infiltrating CD8+ T-cells in the liver biopsy specimen. In two patients, the pathology evaluation during treatment showed dense intratumoral and peritumoral lymphocyte infiltrates, mainly consisting of CD8+ T-cells, which were localized intratumorally. The respective radiological responses at the liver site in these patients were PMD/SD and SMD/PD. Lastly, one patient with SMD as best radiological response at the liver site showed histological response with <10% vital tumor tissue alongside necrosis, fibrosis and inflammation rich in macrophages.

## Discussion

Our study analyzed the radiological response patterns in patients with mUM treated with immunotherapy, including both ipilimumab/nivolumab and tebentafusp. Overall, our results demonstrate that radiological response assessment with RECIST v1.1 and imPERCIST5 is valuable tools for disease evaluation in mUM, but further biomarkers to better stratify patients at risk of disease progression are required. As expected, patients with CR at RECIST were not observed, and CMR at imPERCIST5 was rare. Of note, one patient with CMR as BOR on ipilimumab/nivolumab developed acquired resistance after 12.9 months of response. In a previous phase II study, treatment responses were maintained for a median of 15.6 months (95% CI 1.6–33.8 months) [17]. This indicates that disease relapse in mUM patients with initial treatment response might occur, which is suggestive of acquired treatment resistance and merits further investigation in translational studies. Previous studies indicate that patients with solely extrahepatic disease have a better prognosis when treated with ICIs [16]. Our study included only patients with liver involvement and the overall treatment response was associated with the radiological response at the liver site. This indicates that in patients with mUM and liver involvement, the overall treatment response might be driven by the response at the liver site. The impact of liver-directed therapies in the survival of patients with mUM has been evaluated in retrospective studies in combination with ICIs [22] and tebentafusp [23], with a survival benefit shown in patients treated with ICIs, but not with tebentafusp. Of note, translational studies have shown an extensive intratumoral and intertumoral heterogeneity in UM primary and metastatic liver sites [24, 25], which underlines the disease diversity. Future randomized, prospective studies investigating the combination of immunotherapy in combination with liver-directed treatments, as well as their type and time of administration, are required.

The best overall treatment responses differed between the treatment modalities in the present study, which might be in part attributed to the different mode of action of the two immunotherapy agents. Tebentafusp, an immune-mobilizing monoclonal T-cell receptor, initiates the recruitment and activation of polyclonal T-cells leading to the release of cytokines and other cytolytic mediators to the target cells [26, 27]. In a phase III, randomized clinical trial of tebentafusp compared to investigator's choice in the first-line setting, disease control was achieved in 46% (95% CI 39-52) of patients, whereas the percentage of patients who had an objective response was 9% (95% CI 6-13), with one (0.1%) patient achieving CR and 22 (9%) patients PR as their best response [18]. Despite this low ORR, the 1-year OS rate was 62% (95% CI 53–70%) and the median OS 16.8 months (95% CI 12.9-21.3), which suggests that the radiographic assessment cannot accurately capture the treatment effect. Importantly, in the available liver tumor biopsies in our retrospective study, the histological and the radiological responses were not overall associated. Several biomarkers have been proposed to address this limitation, including immunotherapy-centric criteria, such as the iRECIST [28], tumor growth rate (TGR)-derived parameters [29] and liquid biopsies, including the circulating tumor DNA (ctDNA) [30]. In fact, in an exploratory analysis, early on-treatment reduction in ctDNA was associated with improved OS, even in patients with radiographic progression, thus suggesting that ctDNA can be used as an early indicator of clinical benefit from tebentafusp [31, 32].

Combined treatment with ipilimumab/nivolumab is associated with early tumor shrinkage and prolonged OS in CM [33], but this treatment efficacy could not be confirmed in mUM. In a single-arm, phase II clinical trial of ipilimumab/nivolumab, the ORR was 18% and included one patient with CR and five patients with PR per RECIST v1.1 [16]. Similarly, in a phase II clinical trial at first-line treatment setting, ORR was 11.5% with one patient achieving CR and five patients achieving PR as their best response [17]. In these study protocols, tumor response was evaluated using RECIST v1.1 criteria, which, among other limitations, do not account for tumoral heterogeneity within a lesion and among different lesions at the patient level [34]. Although there was no considerable discrepancy in the assessment of the overall response between RECIST v1.1 and imPERCIST 5.0 criteria in our retrospective study, it can be hypothesized that the latter may outperform the former in assessing the overall tumor response in mUM patients treated with ICIs. Specifically, RECIST v1.1. criteria are not adequate to assess the treatment-induced intratumoral necrosis in liver lesions, which might occur during treatment with ICIs. Another significant factor in radiological assessment with CT is the limitation to differentiate between vital tumor tissue and scar tissue. This represents a significant restriction of RECIST v1.1 criteria, which have been widely adopted in clinical practice as tumor response criteria in solid tumors.

On the other side, the value of FDG-PET/CT in assessing treatment response in metastatic melanoma is not well established yet, despite previous studies showing that FDG-PET can predict long-term outcome in patients treated with immunotherapy in CM [35]. Besides, further studies support the value of FDG-PET/CT to facilitate the differentiation of disease progression from immunotherapy-related radiological patterns, such as pseudoprogression, or immunotherapy-induced sarcoid-like immune reaction [20, 36–40]. As these imaging findings have implications for subsequent treatments and disease prognosis, additional diagnostic tools hold value to readily assess treatment response and resistance in patients treated with immunotherapy. Future studies incorporating on-treatment biopsy specimens with correlative analyses in patients with mUM treated with immunotherapy are required to unravel the dynamic changes of the tumor microenvironment during treatment.

Predictive and prognostic biomarkers to guide treatment selection in patients with mUM, regardless of their HLA-A*02:01 status, are necessary to optimize treatment outcome. Clinical biomarkers, such as time to metastatic diagnosis, presence of bone metastases and LDH level at baseline, were shown to harbor potential prognostic value in patients treated with ICIs [41]. In our retrospective study, elevated LDH at treatment start was associated with worse PFS and OS in patients treated with ipilimumab/nivolumab, albeit without statistical significance. Of note, the OS benefit in patients treated with tebentafusp was independent of the baseline LDH level, which is in accordance with the phase III randomized clinical trial of tebentafusp [18]. In contrast, in a phase II clinical trial of ipilimumab/nivolumab in mUM, elevated LDH of ≥2.5 the upper limit of normal (ULN) at treatment start was associated with shorter PFS (*p*=0.015) and OS (*p*=0.046) [17].

In the available liver tumor biopsies in our retrospective study, the pathological and radiological responses were not overall associated. For instance, in the pathological response assessment of a liver biopsy after treatment with tebentafusp, vital tumor was present in less than 10% of the biopsy specimen and was accompanied by necrosis, fibrosis and inflamed tissue rich in macrophages. The respective radiological response at the liver site per imPERCIST5 was assessed as SMD, thereby reflecting the possible limitation of the current imPERCIST5 criteria. In fact, the diminishing overall metabolism of preexisting tumor tissue through immunologically induced destruction of tumor cells, which can be accompanied by inflamed tissue, may pose a challenge in the radiological response assessment, as inflamed tissues, similar to tumors, tend to uptake increased amount of glucose due to their higher metabolism [42].

Potential limitations of the study should be taken into account when interpreting the findings. Firstly, it should be noted that the number of patients included in the study was relatively small, and that the study was retrospective in nature, thus susceptible to biases. Further, pseudoprogression in imaging under immunotherapeutic treatments is a known phenomenon and could have impacted the results of the radiological response assessment [38, 43]. Lastly, as UM is a rare disease, the study population was very heterogeneous. Nevertheless, it should be recognized that the PFS and OS rates in the present study are comparable to previous publications on ipilimumab/nivolumab and tebentafusp. Furthermore, this is to our knowledge the first study to investigate the value and the limitations of both imPERCIST5 and RECIST v1.1 in treatment response assessment of patients with mUM. Importantly, this real-life patient cohort included patients with at least one prior systemic treatment, which is representative of the clinical reality, since mUM is highly treatment resistant. This detailed evaluation suggests that, similar to CM [36], liver metastases of mUM are associated with poor prognosis, with possibly diverse histological and radiological responses, which underlines the need for further biomarker development. Although both imPERCIST5 and RECIST v1.1 are valuable tools in the radiological response assessment in patients with mUM, the development of accurate biomarkers to stratify the patients at risk for disease progression is highly needed. Future translational studies to investigate specific mechanisms of treatment response and resistance in both treatment agents are required.

## Supplementary Information

Below is the link to the electronic supplementary material.

Supplementary file1 (PDF 414 KB)

## Data Availability

No datasets were generated or analyzed during the current study.
