# Supplementary material for: Immunotherapy response and resistance in patients with advanced uveal melanoma: a retrospective cohort study
Source: Clin Exp Med. 2024 Oct 1;24(1):234. doi: 10.1007/s10238-024-01497-8 (PMC11445343; doi:10.1007/s10238-024-01497-8)
Supplement: Supplementary file 1 — Supplementary file1 (PDF 414 KB) [file 10238_2024_1497_MOESM1_ESM.pdf]

Immunotherapy Response and Resistance in Patients with Advanced Uveal Melanoma; a retrospective cohort study

Alexander Maurer[1*], Giulio Clerici[2*], Jan A. Schaab[1], Phil F. Cheng[3], Daniela Mihic-Probst[4], Cäcilia Mader[1], Michael Messerli[1], Martin W Huellner[1], Reinhard Dummer[2], Florentia Dimitriou[2]

1. Department of Nuclear Medicine, University Hospital of Zurich, University of Zurich, Zurich, Switzerland
2. Department of Dermatology, University Hospital of Zurich, University of Zurich, Zurich, Switzerland
3. Department of Oncology, Geneva University Hospital, Geneva, Switzerland
4. Institute for Pathology and Molecular Pathology, University Hospital Zurich, University of Zurich, Zurich, Switzerland

* Equal contribution as joint first authors

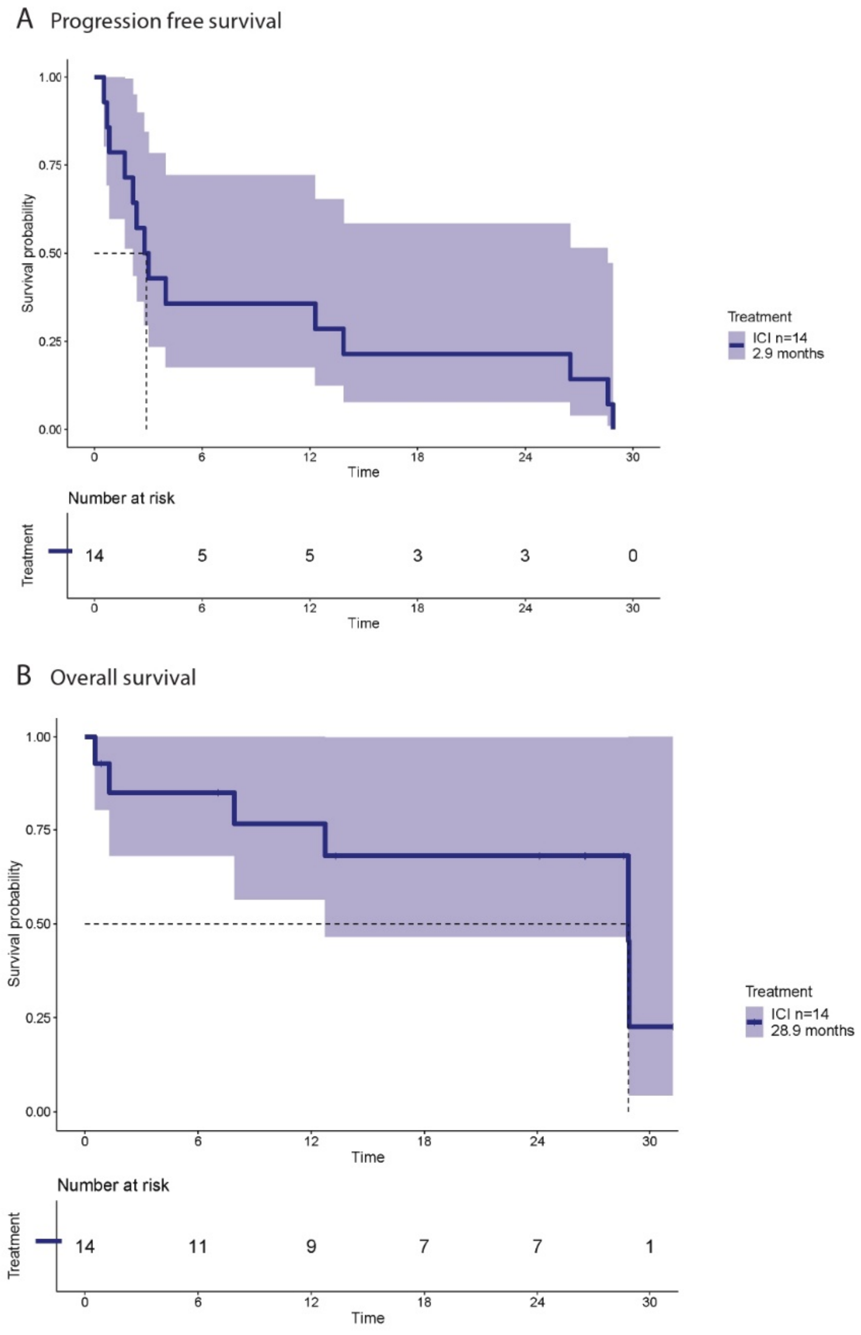

Supplementary Figure 1. Kaplan-Meier curve for progression free survival (PFS) (A) and overall survival (OS) (B) of patients treated with the immune checkpoint inhibitors (ICI) ipilimumab/nivolumab.

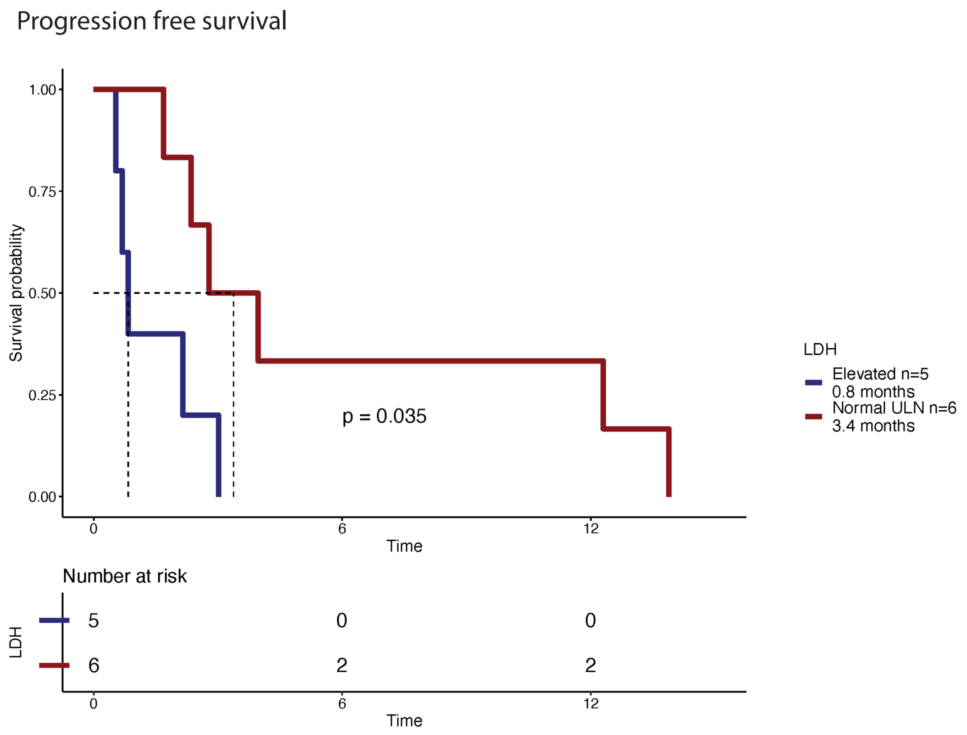

Supplementary Figure 2. Kaplan-Meier curve for progression-free survival (PFS) by lactate dehydrogenase (LDH) level at treatment start with ipilimumab/nivolumab.

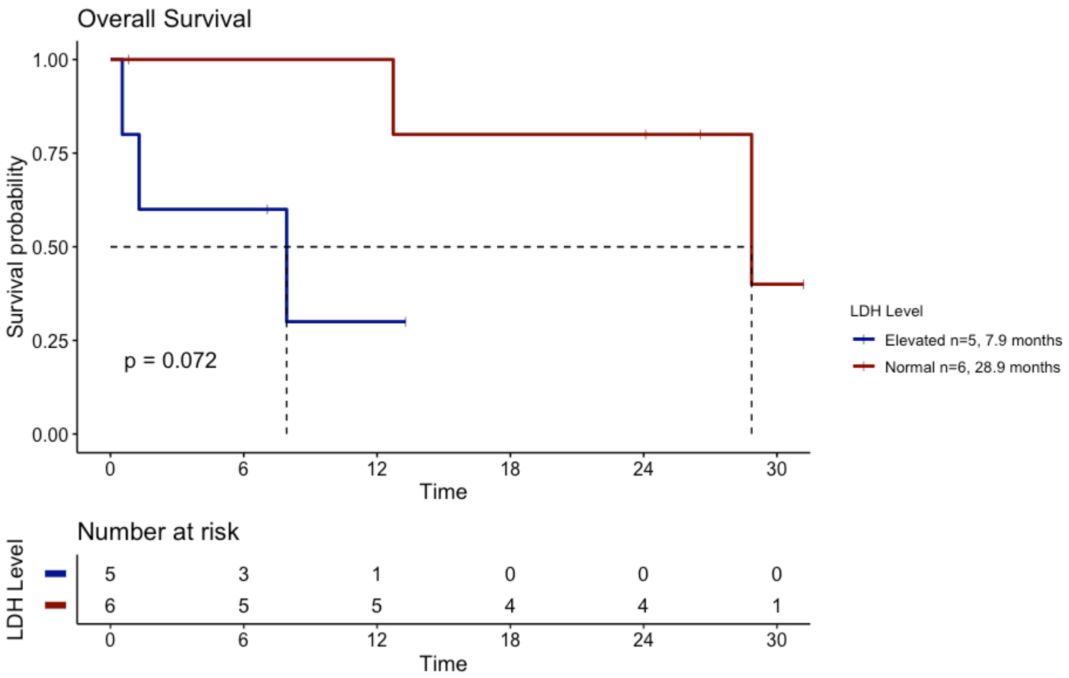

Supplementary Figure 3. Kaplan-Meier curve for overall survival (OS) by lactate dehydrogenase (LDH) level at treatment start with ipilimumab/nivolumab.

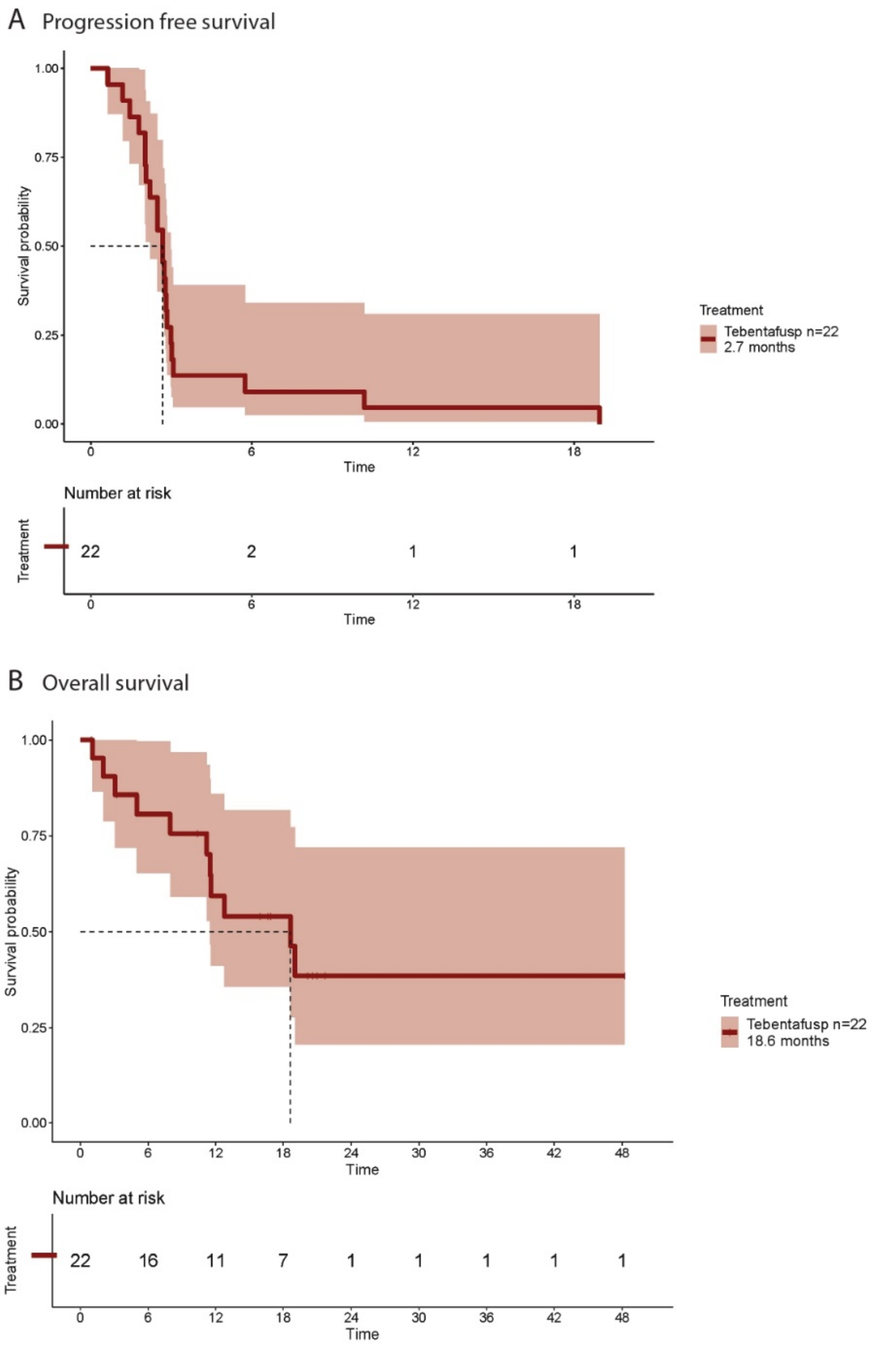

Supplementary Figure 4. Kaplan-Meier curve for progression-free survival (PFS) (A) and overall survival (OS) (B) of patients treated with tebentafusp.

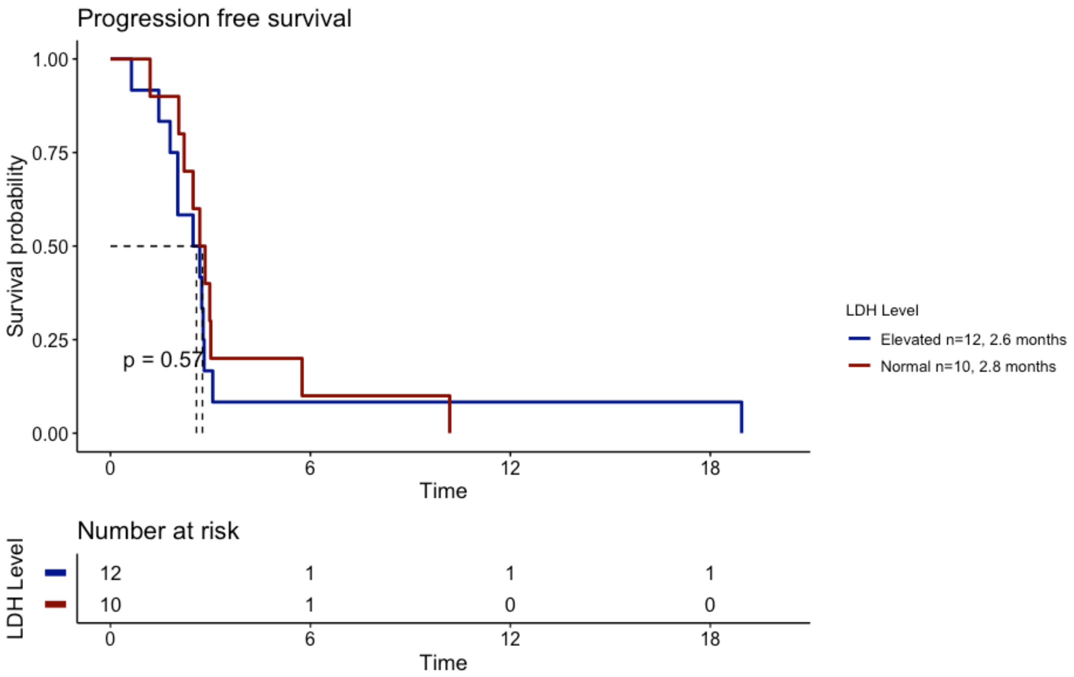

Supplementary Figure 5. Kaplan-Meier curve for progression-free survival (PFS) by lactate dehydrogenase (LDH) level at treatment start with tebentafusp.

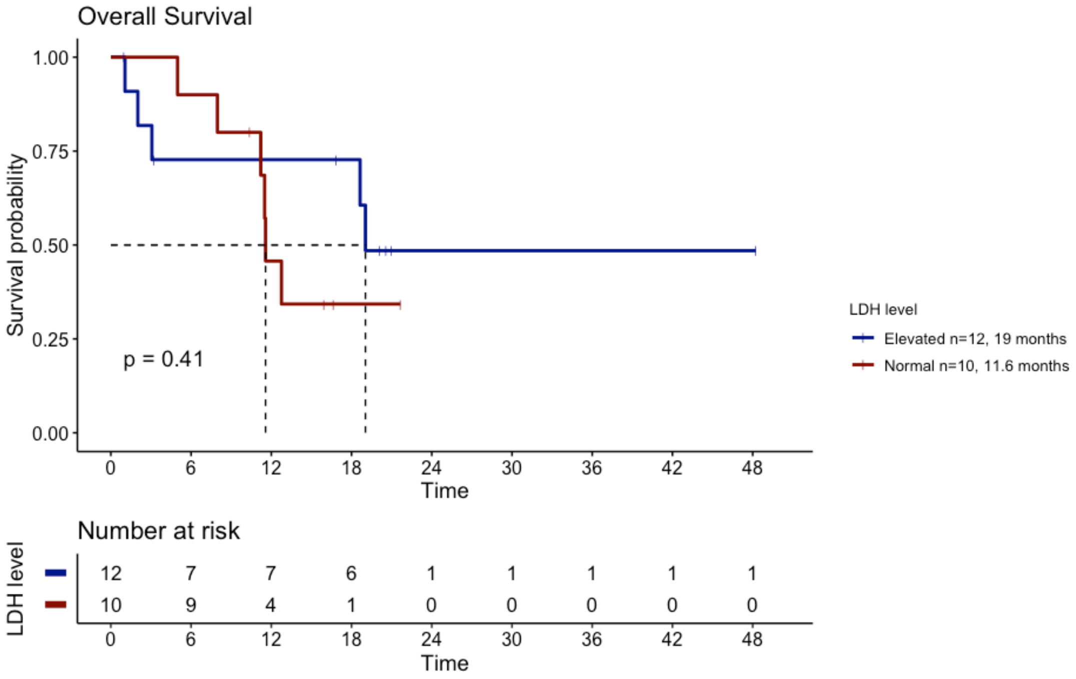

Supplementary Figure 6. Kaplan-Meier curve for overall survival (OS) by lactate dehydrogenase (LDH) level at treatment start with tebentafusp.
